# Microstructure Evolution, Mechanical Properties and Deformation Behavior of an Additively Manufactured Maraging Steel

**DOI:** 10.3390/ma13102380

**Published:** 2020-05-21

**Authors:** Kanwal Chadha, Yuan Tian, Philippe Bocher, John G. Spray, Clodualdo Aranas

**Affiliations:** 1 Planetary and Space Science Centre, University of New Brunswick, Fredericton, NB E3B 5A3, Canada; jgs@unb.ca; 2 Voestalpine Additive Manufacturing Centre Ltd., Mississauga, ON L5N 7Y3, Canada; yuan.tian@voestalpine.com; 3 Department of Mechanical Engineering, Ecole de Technologie Supérieure, Montréal, QC H3C 1K3, Canada; philippe.bocher@etsmtl.ca; 4 Department of Mechanical Engineering, University of New Brunswick, Fredericton, NB E3B 5A3, Canada; clod.aranas@unb.ca

**Keywords:** maraging steel, X3NiCoMoTi18-9-5, laser powder bed fusion, reverted austenite

## Abstract

In this work, the microstructure and mechanical properties of an additively manufactured X3NiCoMoTi18-9-5 maraging steel were determined. Optical and electron microscopies revealed the formation of melt pool boundaries and epitaxial grain growth with cellular dendritic structures after the laser powder bed fusion (LPBF) process. The cooling rate is estimated to be around 10^6^ °C/s during solidification, which eliminates the nucleation of any precipitates. However, it allows the formation of austenite with a volume fraction of about 5% and dendritic structures with primary arm spacing of 0.41 ± 0.23 µm. The electron backscatter diffraction analysis showed the formation of elongated grains with significant low-angle grain boundaries (LAGBs). Then, a solutionizing treatment was applied to the as-printed samples to dissolve all the secondary phases, followed by aging treatment. The reverted austenite was evident after heat treatment, which transformed into martensite after tensile testing. The critical plastic stresses for this transformation were determined using the double differentiation method. The tensile strength of the alloy increased from 1214 MPa to 2106 MPa after the aging process due to the formation of eta phase. The experimental data were complemented with thermodynamic and mechanical properties simulations, which showed a discrepancy of less than 3%.

## 1. Introduction

The additive manufacturing (AM) of metallic materials to form components with complex shapes and geometries is one of the fastest-growing industrial fabrication techniques [1]. The process involves the layer by layer deposition of a material to generate the final product [2]. The major advantages of employing AM technology in the production of metallic parts over conventional manufacturing techniques, such as casting, machining, etc., are the reduction in manufacturing steps and improvement in the utilization of materials [3]. Laser powder bed fusion (LPBF) is one of the most versatile additive manufacturing techniques for metals. In this process, the laser melts a metallic powder, followed by the solidification of the melt to form the final product. One of the limitations of this technique is the crack susceptibility of alloys during solidification [4,5]. Thus, some materials may not be applicable to the LPBF process. Recently, there has been much research on the LPBF of steels [6,7,8,9], nickel-based superalloys [4,5], titanium alloys [10,11], and aluminum alloys [12,13]. Another method similar to LPBF is electron beam melting (EBM) [14]. This process uses an electron beam source instead of a laser beam to melt the metallic powder and form a solid part. Common alloys produced via EBM are steels [15] and titanium alloys [16]. Although EBM can be beneficial due to minimal oxidation and precise control during manufacturing, the focus of the present work will be on LPBF, which is a cheaper and faster manufacturing technique.

Maraging (heat-treated, low-carbon, iron-nickel, martensitic) steels are amenable to AM due to their printability and excellent response to LPBF [17]. The use of AM with this alloy system has facilitated the efficient manufacturing of components deployed in the casting, molding, defense, aerospace, and nuclear industries [18]. Maraging steels require heat treatment to achieve a balance between strength and toughness. This is done by inducing precipitation and secondary phase formation. Although the presence of precipitates is known to increase the tensile strength of maraging steels significantly, the ductility is compromised. The formation of reverted austenite during heat treatment may provide an opportunity to maximize the effects of transformation-induced plasticity (TRIP) [19]. Numerous researchers have studied the formation of reverted austenite and its effects on the final mechanical properties [20,21,22,23,24]. Raabe et al. [20] and Viswanathan et al. [21] presented the benefits of having high austenite volume fraction in maraging steels, accounting for the positive effects on the occurrence of TRIP. However, the existence of austenite could compromise the yield and tensile strengths. Qian et al. pointed out that reverted austenite may not significantly affect some alloys due to the stability of the reverted austenite [22]. Note that these extensive analyses were mostly performed in conventionally manufactured maraging steel.

In this work, following the LPBF process, aging, and tensile testing, the reverted austenite obtained from X3NiCoMoTi18-9-5 maraging steel was measured. For the first time, a possible method to calculate the critical plastic stress to initiate the dynamic phase transformation of reverted austenite to martensite in AM alloy is presented. The current work covers a unique combination of experimental and simulation studies to understand the effect of microstructural evolution during heat treatment on the final mechanical properties of additively manufactured X3NiCoMoTi18-9-5.

## 2. Materials and Methods

**Figure 1:**
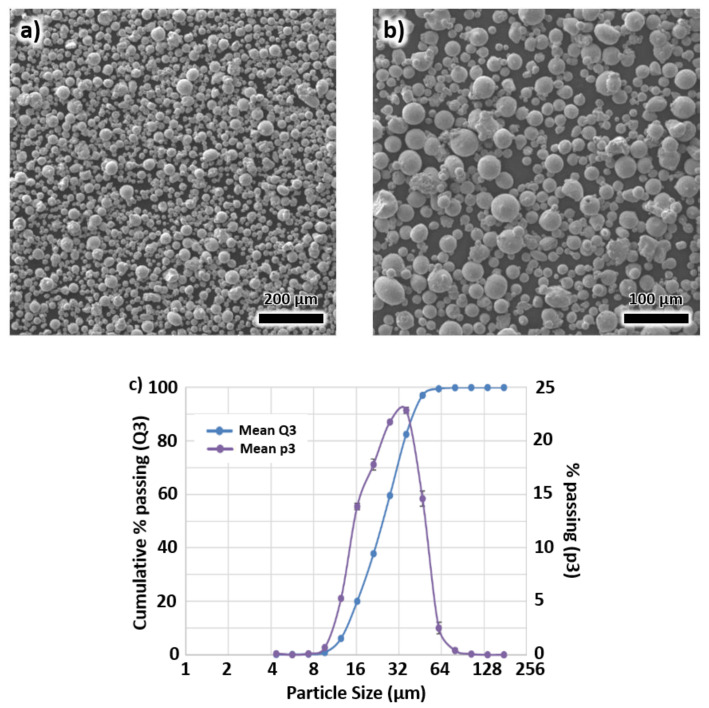
The scanning electron microscopy images of X3NiCoMoTi18-9-5 powders in (**a**) low and (**b**) high magnifications. (**c**) particle size distributions of the powder.

Cubic, rectangular and cylindrical X3NiCoMoTi18-9-5 maraging steel samples were fabricated by voestalpine Additive Manufacturing Center Ltd., Mississauga, Canada (vAMC). These materials were manufactured from gas atomized powders by LPBF utilizing an EOS M290 machine. The maraging steel powder was produced by voestalpine group, with particle diameters ranging from 15 to 45 μm. The composition of the alloy is displayed in Table 1. A scanning electron microscopy image of the powders using the Tescan Vega3 electron microscope is displayed in Figure 1a,b. The particle size distributions of the powder are shown in Figure 1c. The hall flow rate of the powder was tested to be 13.41 s per 50 g.

During the additive manufacturing process, the printing strategy is a zigzag stripe with 50° rotation after every layer, the laser energy density was in the range of 60–70 J/mm^3^, the laser powder was 300 W, the laser speed was 1 cm/s, and the layer thickness was 50 µm. An argon gas atmosphere was employed to minimize oxidation.

Cubic samples (10 mm) were produced for optical and electron microscopy analyses. Then, for tensile testing, cylindrical samples were manufactured with its length parallel to the (a) building direction (BD) and (b) transverse direction (TD), with lengths and diameters of 86 mm and 14 mm, respectively. Finally, samples with 55 mm length and 10 × 10 mm cross-sectional area were fabricated for Charpy impact tests. Similar to tensile tests, two types of sample were created, with lengths parallel to (a) BD and (b) TD. The tensile samples were machined from the cylindrical bars following ASTM E8, with diameters of 6 mm and gauge lengths of 24 mm. For Charpy impact tests, the samples were prepared in accordance with ASTM E23 (V-notch) using an 8 mm striker radius.

The as-printed samples were subjected to solution annealing heat treatment at 850 °C for 1 h, then air-cooled to room temperature. This process was followed by aging at 490 °C for 6 h and air cooling to obtain the final microstructure. All the heat treatment cycles were carried out under argon atmosphere. The as-printed and the heat-treated samples were rough polished using 400, 600, 800, and 1200 grit silicon carbide papers followed by fine polishing using 3 μm and 1 μm diamond suspensions.

For scanning electron microscopy (SEM) imaging, the samples were electro etched by ammonium persulfate solution with an applied voltage of 6 volts for 5 s at room temperature. For the electron backscatter diffraction (EBSD) analysis, the surface of the samples underwent final polishing using a Buehler VibroMet 2 polisher with 0.02 μm silica suspension for 24 h. Electron microscopy was carried out using a Hitachi SU70 equipped with HKL Channel5 software for crystallographic texture analysis. The primary dendrite arm spacing (PDAS) were measured from five different regions using ImageJ image analysis software.

The tensile and Charpy impact tests were carried out along the BD and TD of both as-printed and heat-treated samples in accordance with ASTM E8 (strain rate of 0.002 s^−1^) and ASTM E23, respectively. Five samples for each condition were carried out to verify the results. A C12A Clark hardness tester was used to obtain the hardness measurement of the samples. Thermodynamic and mechanical properties simulations were performed using JMatPro 11.2 software employing the general steel module.

## 3. Results

### 3.1. Microstructure of As-Printed Sample

**Figure 2:**
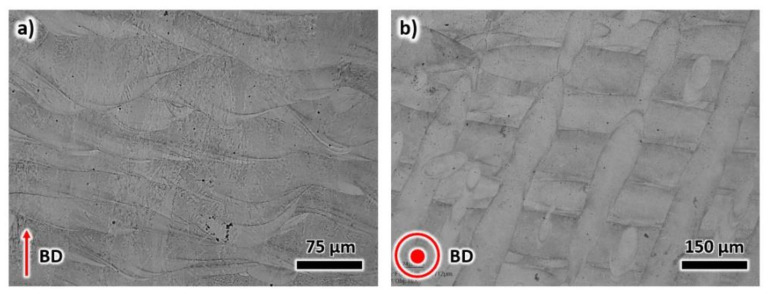
The optical microstructure of the as-printed sample along the (**a**) building direction, and (**b**) transverse direction.

The optical microstructure of the as-printed X3NiCoMoTi18-9-5 maraging steel is displayed in Figure 2a,b. In Figure 2a, the microstructure along the BD shows overlapping melt pool boundaries. The melt pools have an average width (w) and depth (d) of 153 μm and 37 μm, respectively, with an aspect ratio (d/w) in the range of 0.2–0.5. The dimensions of the melt pool are dependent on the processing temperature, scanning velocity, thermal diffusivity, and thermal conductivity [25]. The optical microstructure of the plane normal to the BD, referred here as TD, is presented in Figure 2b. The scan tracks during laser processing are evident, with a thickness of approximately 100 μm.

**Figure 3:**
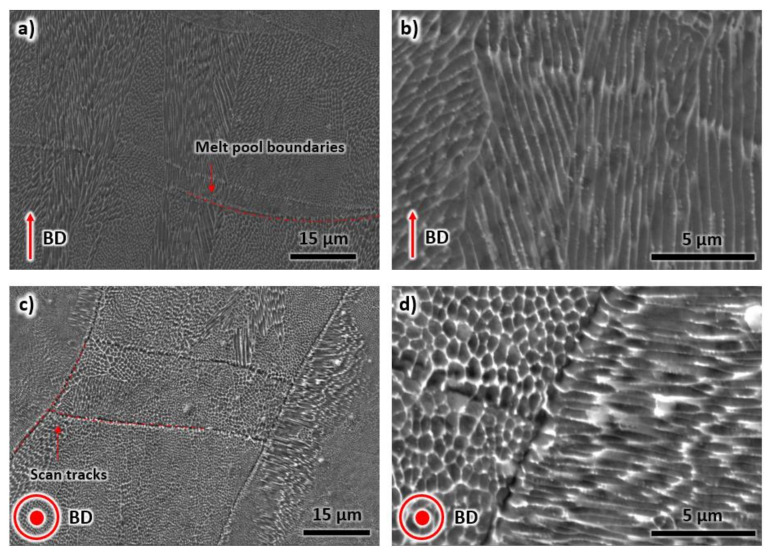
The scanning electron microscopy images of the as-printed samples taken from (**a**,**b**) building direction, and (**c**,**d**) transverse direction.

The SEM images taken from inside the melt pool and scan track are shown in Figure 3. The internal structure of individual melt pools consists of cellular dendritic structures (Figure 3a,b) as a consequence of high cooling rates during LPBF [26,27]. These cellular structures are normally oriented perpendicular to the melt pool boundaries. However, their orientations may deviate due to temperature inhomogeneity during manufacturing, violent Marangoni flow in the melt pool, and crystal orientation [28,29]. The primary dendrite arm spacing was measured to be 0.41 ± 0.23 µm, suggesting the cooling rate in the magnitude of 10^6^ °C/s [7]. The scan tracks from LPBF can be seen in Figure 3c,d. Similar to Figure 3a,b, a cellular structure is also evident; however, the shapes are mostly polygonal rather than dendritic. The cellular polygonally shaped structures appear to be cross-sections of the elongated structures shown in Figure 3a,b.

**Figure 4:**
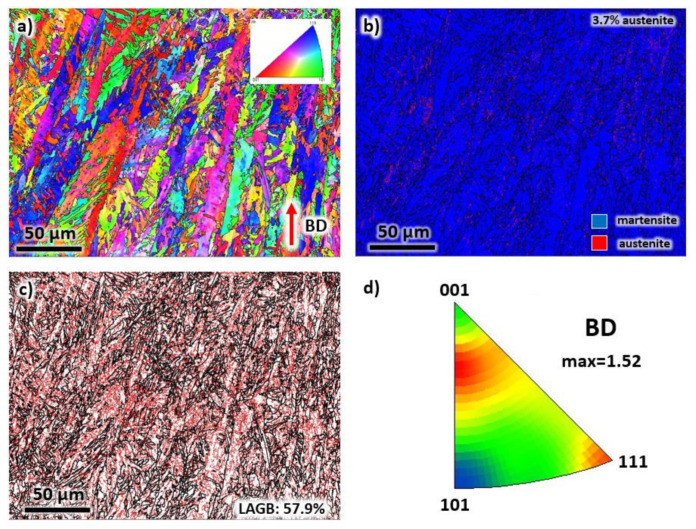
The (**a**) EBSD map, (**b**) phase map, (**c**) grain boundary map, and (**d**) IPF representation of each grain of the as-printed sample taken from the building direction.

An EBSD analysis was performed along the BD to verify the grain morphology and study the crystallographic texture after laser processing. The results are presented in Figure 4. In Figure 4a, the epitaxial growth direction of grains during solidification is confirmed by the elongated grains with widths in the range of 5 to 20 μm. As mentioned above, the growth direction of grains may not be perfectly aligned with the BD. In the present results, the alignment can deviate up to 15°. Another possible reason for the deviation is the positioning of the sample inside the SEM chamber, which can vary by a few degrees with respect to the vertical and horizontal reference axes. Visually, the orientation of the grains does not possess a preferred crystallographic texture, as depicted by the colors of each grain.

The phase analysis of the EBSD map of Figure 4a is presented in Figure 4b. It can be observed that the structure is mostly martensite. However, a volume percentage of 3.7% retained austenite is also present after solidification. The low (LAGBs) and high (HAGBs) angle grain boundaries of the microstructure are presented in Figure 4c. Note that the grain boundaries are defined by HAGBs (misorientation of greater than 15°). On the other hand, the LAGBs is represented by misorientations between 2° to 15° and can be associated with dislocation density inside the material. In the present sample, the solidification is expected to generate significant dislocations in the crystal lattice. As a result, the percentage of LAGBs is relatively high, with a value of 57.9%. The crystal orientation of each grain of the as-printed sample was plotted in an inverse pole figure (IPF) map with reference to BD, see Figure 4c. Although it seems that the structure contained mostly (111) and (001), a low texture intensity factor of 1.52 was measured. Thus, it can be considered that the sample has a random orientation.

**Figure 5:**
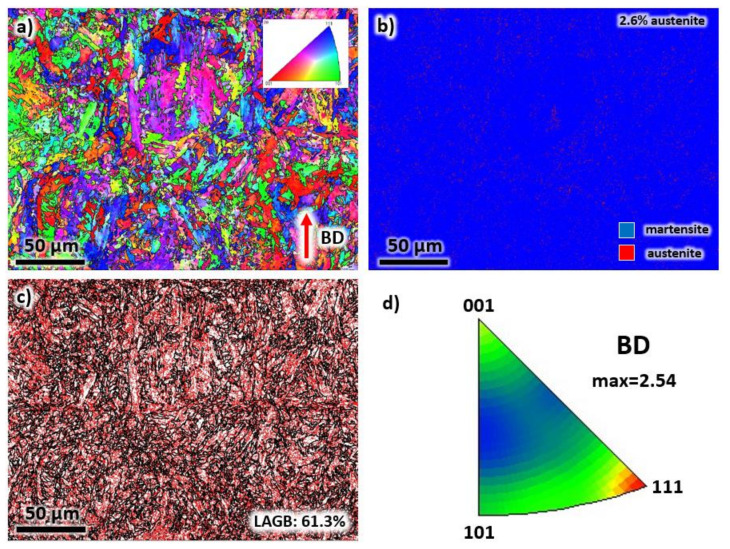
The (**a**) EBSD map, (**b**) phase map, (**c**) grain boundary map, and (**d**) IPF representation of each grain of the as-printed sample taken from the transverse direction.

A similar EBSD analysis presented above was carried out in the TD for the as-printed sample, as illustrated in Figure 5. In Figure 5a, the grains do not have a preferred growth direction, and the morphology is mostly polygonal. These grains are the cross-sectional areas of the elongated grains in the microstructures of BD. The phase contains mainly martensite with a minor amount of retained austenite (2.6%), see Figure 5b. In Figure 5c, the LAGBs is comparable to that of the BD, with a volume fraction of 61.3%, which confirmed a relatively high dislocation density of the sample after solidification. The crystallographic texture displays a high intensity of (111) in IPF representation with BD as a reference axis (see Figure 5d). However, the texture intensity is still relatively low, measured at 2.54. This number confirms that the crystal orientations are randomly arranged after the LPBF process.

**Figure 6:**
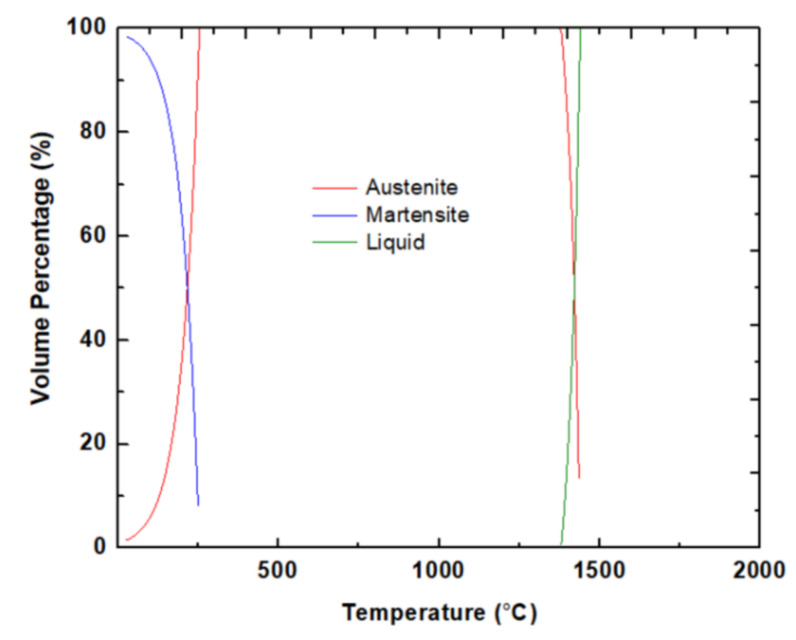
The expected phases of the sample during solidification at a cooling rate of 10^6^ °C/s.

A thermodynamic simulation was performed to confirm the phases after solidification using a cooling rate of 10^6^ °C/s. The solidification function of the JMatPro 11.2 software employing the general steel module was performed, and the result is displayed in Figure 6. Similar to experimental results, the microstructure is mostly martensite without any precipitation. Here the martensitic start temperature is approximately 250 °C. Above 250 °C, the phase is austenitic. Note that these phases are based on cooling from the liquid phase at a cooling rate of 10^6^ °C/s. At equilibrium, the fraction of phases (and precipitates) is significantly different. After cooling, the expected retained austenite is 1.45%. In the present sample, the retained austenite is 3.7% and 2.6% for BD and TD, respectively. These numbers are within the experimental error when measuring the volume fraction of phases. Thus, based on the simulation, it appears that the experimental observations are quite reasonable.

### 3.2. Microstructure of Heat-Treated Sample

**Figure 7:**
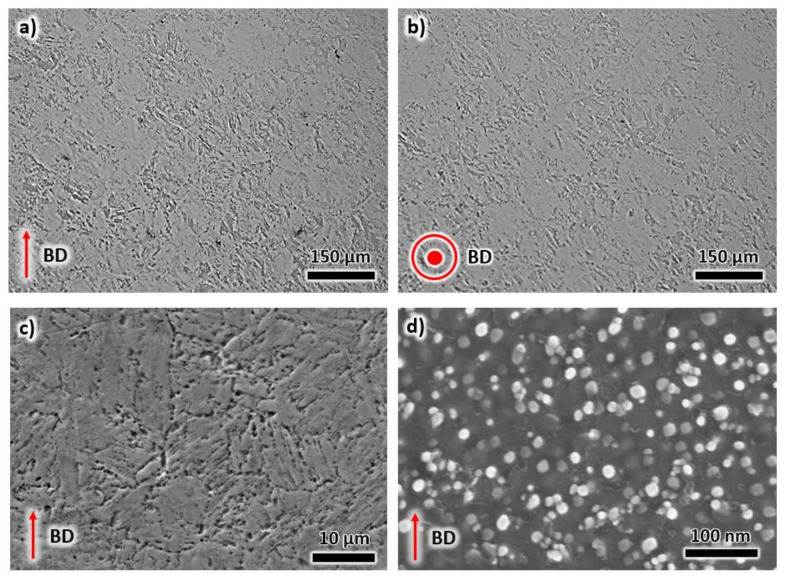
Optical microstructures of the heat-treated sample taken from (**a**) building direction, and (**b**) transverse direction. SEM images of the same sample using (**c**) low magnification and (**d**) high magnification imaging.

Solution annealing and aging heat treatment cycles following printing resulted in the disappearance of melt pool boundaries and scan tracks, as shown in the micrographs of Figure 7. The heat treatment process eliminated the fusion zone between layers during LPBF (see Figure 7a,b), which assists in homogenizing the mechanical properties of the alloy along the BD and TD. Here, the microstructure of the heat-treated sample can be compared to a conventionally fabricated maraging steel, as shown in the SEM image in Figure 7c [30]. A martensitic needle-like structure is evident. The microstructure also contains precipitates with sizes of less than 10 nm, as illustrated in the high-magnification SEM image in Figure 7d. The presence of precipitates was validated in the thermodynamic simulation in Section 4.1.

**Figure 8:**
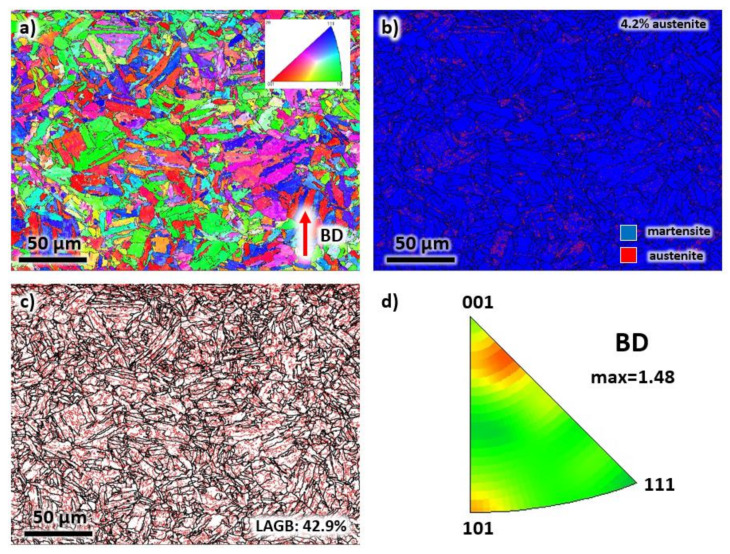
The (**a**) EBSD map, (**b**) phase map, (**c**) grain boundary map, and (**d**) IPF representation of each grain of the heat-treated sample taken from the building direction.

The morphology of grains significantly changed after the annealing and aging heat treatment, as confirmed by the EBSD analysis in Figure 8. The elongated grains in Figure 4 transformed into a mixture of polygonal and plate-like martensitic structures (see Figure 8a), similar to the microstructure of a conventionally manufactured maraging steel [31]. By observation, the distribution of the colors of the grains indirectly suggests a random orientation of grains. Additionally, the microstructure contains 4.2% of the reverted austenite phase produced after aging (see Figure 8b). Note that the initial solutionizing treatment dissolves the secondary phases and precipitates while the aging treatment assists in the formation of secondary phases and precipitates. The volume fraction of LAGBs decreased to 42.9%, as presented in Figure 8c due to the annihilation of dislocations at elevated temperatures. In Figure 8d, similar to the as-printed sample, although (001) and (101) planes consist of the majority of the grain orientations, a weak crystallographic texture is observed with a maximum texture intensity factor of 1.48.

**Figure 9:**
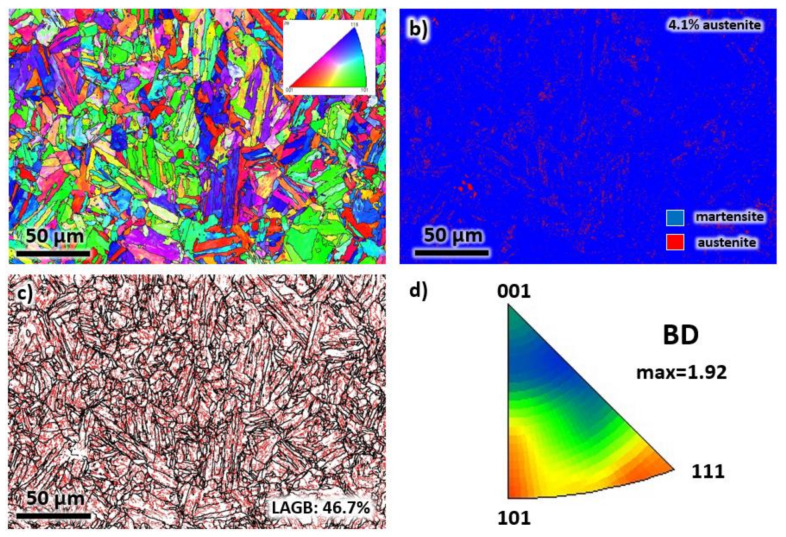
The (**a**) EBSD map, (**b**) phase map, (**c**) grain boundary map, and (**d**) IPF representation of each grain of the heat-printed sample taken from the transverse direction.

An EBSD analysis was also carried out on the heat-treated sample in the TD, see Figure 9. In Figure 9a, it can be deduced that the grain morphology is comparable to the BD direction. A mixture of polygonal and plate-like martensitic structures was observed. The sample is mostly martensitic, with 4.1% reverted austenite (see Figure 9b). The LAGBs decreased to 46.7%, as shown in Figure 9c. For the crystallographic texture (see Figure 9d), although most of the grains have orientations of (111) and (101) with reference to the BD, the texture intensity factor is small. Thus, it can be concluded that the sample has randomly oriented grains. Note that the observations taken from the EBSD analysis in the TD are comparable with the results obtained from the BD in Figure 8.

## 4. Discussion

### 4.1. Mechanical Properties

**Figure 10:**
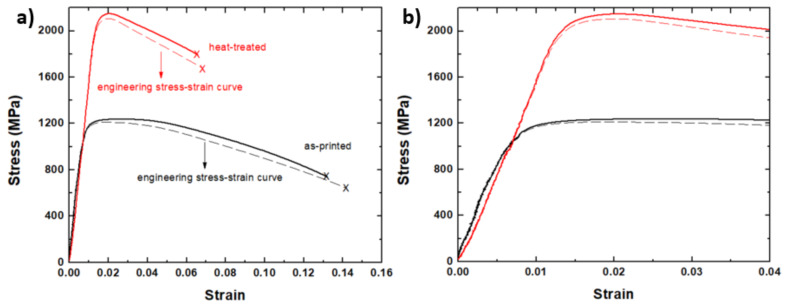
(**a**) The stress-strain curves of the as-printed and heat-treated samples. (**b**) A magnified view of the stress-strain curves in Figure 10a.

The engineering stress-strain curves associated with the tensile tests of the as-printed (broken black line) and heat-treated (broken red line) samples along the BD are displayed in Figure 10a. A representative from five measurements was shown here since the measurements have less than 3% difference. This information was utilized to obtain the mechanical properties data such as tensile strength, yield strength, and elongation. These engineering stress–strain curves were then converted into true stress–strain curves, as presented in solid lines, to account for the instantaneous change in the cross-sectional area during deformation. The average ductility of the as-printed sample (15%) is significantly higher than the heat-treated sample (8%). Moreover, the toughness of the as-printed specimen (59 J) also decreased to 11 J after heat treatment. On the other hand, its average tensile strength (1212 MPa) is considerably lower than the heat-treated sample (2105 MPa). Similarly, the average hardness of the as-printed sample (35 HRC) is significantly lower than the heat-treated sample (53 HRC). The complete mechanical properties of the alloy obtained from this work are summarized in Table 2. Note that the standard deviation was calculated based on five measurements. This change in mechanical properties is mainly due to the formation of precipitates during the aging process. In the present alloy, precipitates and reverted austenite are both present in the microstructures, as illustrated above.

**Figure 11:**
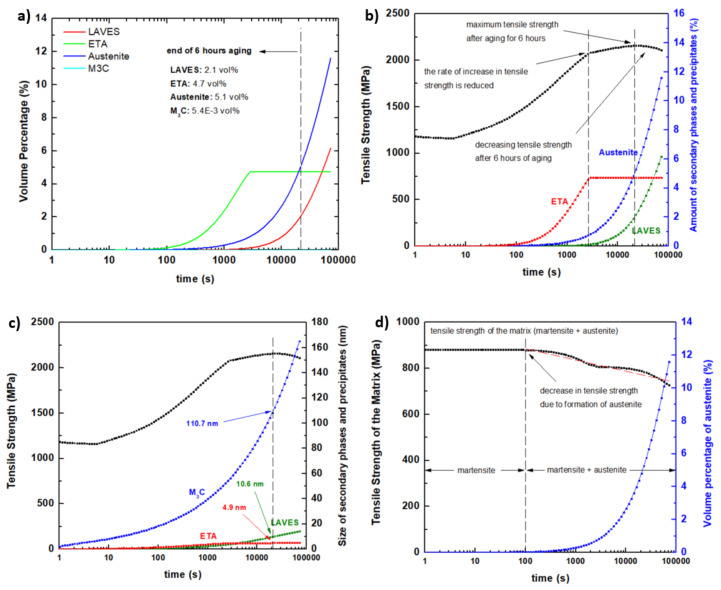
The dependence of (**a**) volume fraction of precipitates and secondary phases on aging time. The effects of (**b**) the type of phases, and (**c**) size of the precipitates on tensile strength during the aging process. (**d**) The impact of increasing reverted austenite on the tensile strength.

A thermodynamic simulation was performed using the solutionizing and aging heat treatment cycle to determine the volume fractions of precipitates and secondary phases, including their effect on tensile strength. The simultaneous precipitation of the maraging steel function of JMatPro was employed. The results are presented in Figure 11. The solution annealing and air cooling convert the microstructure to a fully austenitic phase, which eventually transforms into a martensitic phase during cooling. The aging treatment at 490 °C may produce various precipitates, intermetallics, and other phases, as displayed in Figure 11a. Before aging, the microstructure was fully martensitic; however, after isothermal holding for 6 h, the following were formed: (i) laves phase with 2.1 vol%, (ii) eta phase with 4.7 vol%, (iii) reverted austenite phase with 5.1%, and (iv) M3C with 0.005 vol%. Note that the reverted austenite was measured to be about 4.2 vol% in the EBSD images of Figure 8b and Figure 9b. Thus, from a microstructural point of view, it appears that the values from experiments and simulations are comparable.

The effect of eta, laves, and austenite on tensile strength is illustrated in Figure 11b. The tensile strength is observed to increase rapidly during aging as the volume fraction of eta is increased. The rate of increase in tensile strength is reduced (as depicted by the slope of the curve) after the volume fraction of eta reached a maximum value of 4.7 vol% at approximately 3000 s of aging time. At this point, the tensile strength increased from 1185 MPa (before aging) to 2090 MPa. Based on this information, it appears that the main strengthening of the alloy is the presence of eta precipitates. After 3000 s, the tensile strength continued to increase at a slower rate due to the formation of the laves phase. However, the increasing amount of austenite prevents any significant increase in tensile strength. Thus, from 3000 s to 20,000 s of aging time, the tensile strength only increased from 2090 MPa to about 2160 MPa. After 20,000 s of aging time, a decreasing tensile strength is observed. The critical amount of reverted austenite, which causes a reduction in tensile strength, is shown to be 5.1 vol%. Above this volume percentage, it is expected that the softer austenite phase will have a significant impact on the overall mechanical properties of the alloy.

The size of M3C, eta, and laves phases was simulated in Figure 11c. After the aging cycle, the predicted sizes of the M3C, eta, and laves are 110 nm, 4.9 nm, and 10.6 nm, respectively. Based on these numbers, it appears that the precipitates in the SEM image of Figure 7d are mainly eta precipitates. A more detailed analysis of these precipitates will be a part of the future study of the present researchers.

The effect of the increasing amount of reverted austenite on the tensile strength of the matrix (martensite and austenite) without any precipitates is illustrated in Figure 11d. This information validates the argument in Figure 11b. Here, it can be seen that the strength of the matrix immediately decreases as soon as the reverted austenite started to form at an aging time of about 90 s. From 100 s to 100,000 s of aging time, the presence of reverted austenite reduces the tensile strength of the alloy by approximately 15 MPa per 1 vol%.

Note that, in the mechanical properties simulation above, the tensile strengths of as-printed and heat-treated samples are 1185 MPa and 2160 MPa, respectively. The experimental values are 1212 MPa and 2105 MPa for as-printed and heat-treated samples, respectively. The discrepancy between the simulation and experimental data is less than 3%. The results in this work clearly show a close agreement between the experimental and simulation work.

### 4.2. Analysis of the Deformed Sample

**Figure 12:**
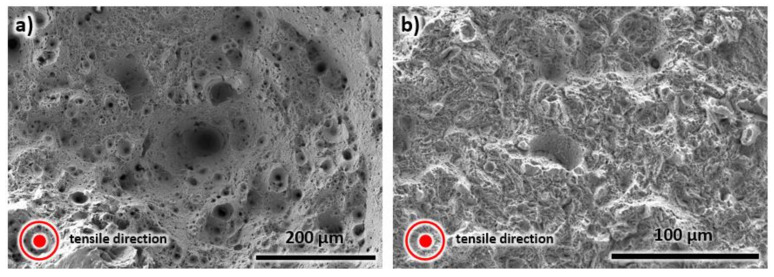
The SEM images of the fractured surface of (**a**) as-printed, and (**b**) heat-treated sample.

A room temperature tensile test was performed on both as-printed and heat-treated samples. The SEM images of the fractured surface of the as-printed and heat-treated samples are presented in Figure 12. These images have an apparent difference. A typical microstructure for ductile failure with a distinct dimple microstructure can be observed in the as-printed sample (see Figure 12a). On the other hand, Figure 12b illustrates a brittle failure as denoted by cleavage fracture. The difference between the samples is consistent with the mechanical properties stated above. The as-printed specimen has a lower tensile strength but higher ductility than the heat-treated sample. Thus, the former shows a dimple structure, while the latter displays a cleavage fracture.

**Figure 13:**
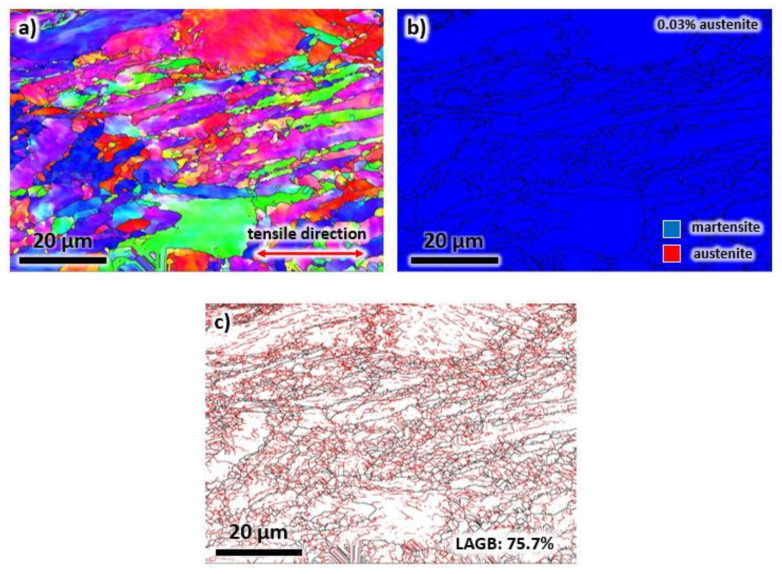
The (**a**) EBSD map, (**b**) phase map, and (**c**) grain boundary map of the heat-printed sample after tensile testing taken from the building direction.

The orientation and shape of the grains after tensile testing of the heat-treated sample were assessed using the EBSD technique (Figure 13). As expected, the grains are elongated along the direction of the tensile stress, as depicted in Figure 13a. Interestingly, following heat treatment, all the austenite phase has been transformed into martensite as a result of applied stress (see Figure 13b). Moreover, the volume fraction of LAGBs increased to 75.7%, as displayed in Figure 13c. This increase is due to the dislocations generated during room temperature deformation. Note that this value is higher than the volume fractions of LAGBs in the as-printed and heat-treated samples.

Although the precipitates provide a significant increase in strength and hardness in maraging steels, the present work attempted to provide an in-depth analysis of the behavior of reverted austenite during deformation. The austenite phase at room temperature is known to offer a good combination of strength and ductility via activating the mechanism of transformation-induced plasticity [32]. The idea is to obtain and identify the critical plastic stress to induce the dynamic phase transformation in the present material.

As observed from the EBSD images, the reverted austenite has been completely transformed into martensite after straining. This phase transformation is a deformation-induced phenomenon and takes place dynamically. Therefore, in this work, the critical plastic stress to induce the transformation of austenite to ferrite is assumed to be in the plastic region, more specifically between the yield and peak stresses. The magnified image of the stress-strain curve in Figure 10b indicates the location of the 0.2% offset yield and peak stresses. Strain hardening occurs in this region, and the strain hardening rate changes due to various hardening and softening metallurgical phenomena [33]. In this work, a softer phase (austenite) transforms into a harder phase (martensite). Thus, an increase in the strain hardening rate is expected.

The double differentiation method [34,35,36,37] was applied to the true stress-strain curves of Figure 10a to detect the change in the hardening rate during deformation. This technique is commonly utilized in hot deformation studies to track the evolution of microstructures and fluctuations in strain hardening rate [21,22]. The present work is the first attempt to apply such a method to the mechanical test results obtained from an additively manufactured alloy to detect the initiation of a hardening mechanism.

**Figure 14:**
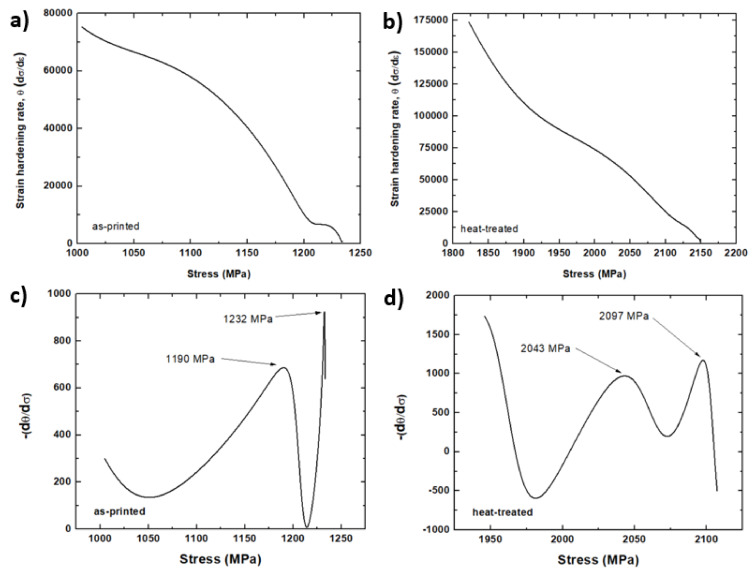
The dependence of strain hardening rate on applied stress for (**a**) as-printed and (**b**) heat-treated samples. The dependence of the derivative of strain hardening rate on the applied stress for (**c**) as-printed and (**d**) heat-treated samples.

The true stress–strain curves of the as-printed and heat-treated samples were fitted with 9th order polynomial equations using Matlab software. Then, the dependence of strain hardening rate at a fixed strain rate, *θ* = (*δσ*/*δε*)*_ε̇_*, with applied stress is measured, as illustrated in Figure 14a,b for as-printed and heat-treated samples, respectively. The inflection point of the *θ-σ* curve can be associated with hardening or softening mechanisms denoted by:

(1) $$\frac{\delta }{\delta \sigma }\left(\frac{\delta \theta }{\delta \sigma }\right)=0$$

The inflection points were tracked by obtaining the dependence of −*δθ*/*δσ* on applied stress, as displayed in Figure 14c,d for the as-printed and heat-treated samples, respectively.

The maxima of the −*δθ*/*δσ* versus the applied stress determine any possible hardening mechanisms. In Figure 14c, the first peak is equal to 1190 MPa, a stress value comparable with the measured yield stress of 1135 MPa. The peak corresponds to the change in strain hardening rate as a result of reaching the yield point during deformation. Note that the work hardening in the plastic region is significantly different than that of the plastic region of the stress–strain curve. The second peak has a stress value of 1232 MPa, which probably associated with the transformation of reverted austenite into martensite. This critical plastic stress value is between the yield and peak stresses. This peak may also be due to other metallurgical phenomena, which remains to be confirmed experimentally. Similarly, in Figure 14d, the first peak is 2043 MPa, which is comparable with the measured yield stress of the heat-treated sample (2055 MPa). On the other hand, the second peak is measured at 2097 MPa. Once again, this critical plastic stress value is between the yield and peak stresses. This observation appears to be related to the deformation-induced phase transformation of reverted austenite to martensite, which needs further investigation. Thus, based on the assumption in this work, the phase transformation critical plastic stresses for the as-printed and heat-treated samples are 1232 MPa and 2097 MPa, respectively.

## 5. Conclusions

In summary, this work has revealed elongated grains with nano-scale cellular dendritic structure in X3NiCoMoTi18-9-5 maraging steel after the laser powder bed fusion process. This microstructure transformed into a martensitic lath microstructure after heat treatment. The thermodynamic calculations suggest that retained austenite was formed during solidification after laser processing, which is consistent with the EBSD analysis of the as-printed sample. The retained austenite completely transformed into martensite after solution annealing heat treatment. However, the aging process produced reverted austenite. These observations agree with the thermodynamic simulation of the heat treatment cycle. The main strengthening appears to be the presence of eta precipitates until 3000 s of aging time. After 3000 s, it seems that the laves phase slightly increases the tensile strength until 20,000 s. After 20,000 s, a critical amount of reverted austenite (5.1 vol%) is reached, which results in a reduction in tensile strength. The presence of reverted austenite reduces the tensile strength of the alloy by approximately 15 MPa per 1 vol%. The reverted austenite was shown to transform during tensile testing. For the first time, a method to calculate the critical plastic stress of a hardening mechanism in an additively manufactured alloy was presented. Based on the assumption in this work, the critical plastic stresses associated with transformation-induced plasticity in X3NiCoMoTi18-9-5 maraging steel are 1232 MPa and 2097 MPa for as-printed and heat-treated conditions, respectively.

## Figures and Tables

**Table 1:** The complete chemical composition of X3NiCoMoTi18-9-5 steel (in wt %).

C	Si	Mn	Mo	Ni	Co	Ti	Fe
0.03	0.10	0.15	4.90	18.00	9.30	1.10	balance

**Table 2:** Mechanical properties of X3NiCoMoTi18-9-5 maraging steel.

	As-Built (BD)	As-Built (TD)	Heat-Treated (BD)	Heat-Treated (TD)
Tensile Strength	1214 ± 3 MPa	1215 ± 5 MPa	2106 ± 2 MPa	2121 ± 8 MPa
Yield Strength	1135 ± 4 MPa	1155 ± 4 MPa	2055 ± 11 MPa	2058 ± 10 MPa
Elongation	15 ± 2%	15 ± 2%	8 ± 2%	8 ± 2%
Hardness	35 ± 1 HRC	35 ± 1 HRC	53 ± 3 HRC	53 ± 3 HRC
Toughness	59 ± 1 J	59 ± 1 J	11 ± 2 J	11 ± 2 J

## Nomenclature

AM	additive manufacturing
ASTM	America Society for Testing and Materials
BD	building direction
EBSD	electron backscatter diffraction
HAGBs	high-angle grain boundaries
IPF	inverse pole figure
LAGBs	low-angle grain boundaries
LPBF	laser powder bed fusion
PDAS	primary dendrite arm spacing
SEM	scanning electron microscopy
TD	transverse direction
TRIP	transformation-induced plasticity
